# Supplementary material for: A country-wide health policy in Chile for deaf adults using cochlear implants: Analysis of health determinants and social impacts
Source: PLoS One. 2023 Oct 25;18(10):e0286592. doi: 10.1371/journal.pone.0286592 (PMC10599544; doi:10.1371/journal.pone.0286592)
Supplement: S2 File — (DOCX) [file pone.0286592.s002.docx]

**A country-wide health policy in Chile for**  **deaf adults using cochlear implants: analysis of health determinants and social impacts**

Mario Bustos-Rubilar^1,2,*^, Fiona Kyle^1^, Eliazar Luna^3^, Karim Allel^4^, Ximena Hormazabal*^5^*, Daniel Tapia-Mora*^26^*, Merle Mahon^1^

*^1^ Division of Psychology and Language Science, University College London, London, UK*

*^2^ Departamento de Fonoaudiología, Facultad de Medicina, Universidad de Chile, Santiago, Chile.*

*^3^ Institute of Epidemiology and Healthcare, University College London, London, UK*

*^4^ Institute for Global Health, University College London, London, UK*

*^5^ Carrera de Fonoaudiología, Pontificia Universidad Católica de Chile, Santiago, Chile.*

*^6^ Escuela de Fonoaudiología, Universidad de los Andes, Santiago, Chile.*

^*^ **Corresponding author**. Division of Psychology and Language Sciences, Brain Faculty, University College London, 2 Wakefield St, London WC1N 1PJ, email: mario.rubilar.18@ucl.ac.uk

Table of Contents

S1 Appendix- Protocols:

[1 INFORMED CONSENT FOR ADULTS USING CI 1](#_Toc121247030)

[2. INFORMED CONSENT FOR ADULTS USING CI 2](#_Toc121247031)

[3. ENGLISH PROTOCOL FOR ADULTS USING CI (Short version) 4](#_Toc121247032)

[4. SPANISH PROTOCOL FOR ADULTS USING CI (Extended version with IOI-HA assessment) 6](#_Toc121247033)

S2 Appendix – Consort diagram

**S1 Appendix –‘Protocols’**

## INFORMED CONSENT FOR ADULTS USING CI (ENGLISH SHORT VERSION)

We need your consent for doing this online survey. The purpose of this information is to help you decide to participate or not - in the research: "**Characterisation of Cochlear Implant Users in Chile ".**

**The research objective** is to characterise the Chilean cochlear implant users from Public System Health implanted from 2017.

If you agree to participate, we will invite you to take part in an interview to ask you about you and your CI. The interview will be either face-to-face or on the telephone and will take about 20 minutes. We will collect information about you, your family, your cochlear implant, your treatment and use of the device

The information collected will be confidential and pseudonymised, according to Chilean and British law (GPDR) about protecting user data. It means that real names will not be used, and this interview will have an ID number. Nobody in your Hospital will know any answer you give today. Only the research team will have access to the data.

The data will be analysed at University College London in the UK. All data transferred to the UK will be fully anonymised, and no personally identifiable data will be transferred to the UK.

**About benefits** – The results obtained will be useful to our understanding of the current situation in all users implanted in the public system.

If required, at the end of the study, we can send you a summary report of the study results.

**About risks** - This research has no harmful effects.

**About compensations** - As this study is not funded, we are unable to offer any financial compensation for the time you spend taking part.

**This will be voluntary participation** - Your participation in this research is entirely voluntary. You can withdraw at any time without giving a reason. If you decide to withdraw you will be asked what you wish to happen to the data you have provided up that point.

It is up to you to decide whether or not to take part. If you choose not to participate, you won't incur any penalty or loss of benefits to which you are otherwise entitled as a patient at the Hospital. Refusing will not affect the quality of care you receive.

**Questions** - If you have questions about your rights as a participant in medical research, you can follow the link that we will send you through e-mail with this consent and other details about this project. At the same time, if you don't have e-mail, we will advise you at the end of this consent about the link in the internet and other ways that we have for giving all the information that you need.

The Ethics Committee from Faculty of Medicine of the University of Chile and University College of London have approved this study

**Name and Contact Details of the Researcher(s):**

The principal research is Mario Bustos Rubilar, his mail [mario.rubilar.18@ucl.ac.uk](mailto:mario.rubilar.18@ucl.ac.uk) telephone

**Do you understand what we are asking you to agree to? ____ Yes _____ No**

**Do you agree to participate in this interview? ____ Yes _____ No**

**What is your name? ____________________________________________________.**

## 2. INFORMED CONSENT FOR ADULTS USING CI – SPANISH VERSION

Usted ha sido invitado/invitada a participar en este estudio. El propósito de esta información es ayudarlo a decidir si participa o no en la investigación: "Caracterización de los usuarios de implantes cocleares en Chile" del Ministerio de Salud Chileno, la University College of London y la Universidad de Chile. El objetivo de la investigación es caracterizar a los usuarios chilenos de implantes cocleares del Sistema Público de Salud implantados a partir de 2017.

Si acepta participar, le invitamos a completar este cuestionario respecto a usted y su Implante Coclear. En el caso que no pueda completar la entrevista en esta plataforma, dispondremos de un servicio telefónico y/o de interpretación en lengua de señas que podrá guiarlo en este proceso. La duración de esta actividad es aproximadamente 30 minutos.

La información recopilada será confidencial y pseudo anonimizada, de acuerdo con la ley chilena y británica (GPDR) sobre protección de datos de los usuarios. Esto significa que no se usarán nombres reales y este cuestionario tendrá un número de identificación. Nadie en su Hospital sabrá la respuesta que dé hoy. Solo el equipo de investigación tendrá acceso a los datos. Los datos se analizarán en la University College London en el Reino Unido. Todos los datos transferidos al Reino Unido serán completamente anonimizados y no se transferirán datos de identificación personal a ese país.

Adicionalmente, esta investigación utiliza algunos datos provenientes desde la historia clínica de los usuarios con Implante Coclear, los cuales son anónimos para proteger su información personal.

Acerca de los beneficios: Los resultados obtenidos serán útiles para comprender la situación actual de todos los usuarios implantados en el sistema público. Si es necesario, al final del estudio, podemos enviarle un informe resumido de los resultados del estudio.

Acerca de los riesgos: esta investigación no tiene efectos dañinos.

Acerca de las compensaciones: No podemos ofrecer ninguna compensación financiera por el tiempo que dedique a participar.

Esta será una participación voluntaria: su participación en esta investigación es completamente voluntaria. Puede retirarse en cualquier momento sin dar una razón. Si decide retirarse, se le preguntará qué desea que suceda con los datos que ha proporcionado hasta ese momento.

Depende de usted decidir si participa o no. Si elige no participar, no incurrirá en ninguna sanción ni perderá los beneficios a los que tiene derecho como paciente del Hospital. Negarse no afectará la calidad de la atención que reciba.

Preguntas: Si tiene preguntas sobre sus derechos como participante en una investigación médica, puede comunicarse con el investigador principal de este estudio, cuyos datos se encuentran al final de este consentimiento. A su vez, si no puede pero desea completar este cuestionario/consentimiento, nos comunicaremos con usted por teléfono y recolectaremos información vía grabación de voz, la cual quedará almacenada digitalmente junto a sus datos personales.

Este estudio ha sido registrado por en Reino unido por UCL Legal Services con el código Nº Z6364106/2020/06/92. Al mismo tiempo, este consentimiento ha sido aprobado por el Comité de Ética de la Facultad de Medicina de la Universidad de Chile.

Para  comunicarse con el comité de ética de investigación en seres humanos (CEISH):  Teléfono: 22 978 9536 , Correo electrónico: comiteceish@med.uchile.cl - [ceish.uchile@gmail.com](mailto:ceish.uchile@gmail.com) Universidad de Chile en Av. Independencia 1027, Independencia

El investigador principal es Mario Bustos Rubilar. Si necesita ponerse en contacto con él su teléfono es 229786606 y su correo es mario.rubilar.18@ucl.ac.uk

¿Acepta participar en este estudio?

SI __ NO___

¿Cuál es tu nombre? ____________________________________________________.

Consideraciones para el Consentimiento informado a padres/cuidadores a través de OPINIO Platform o Entrevista telefónica:

- Este consentimiento estará disponible en la platforma online OPINIO (se han adjuntado los link para el análsis de la plataforma de cuestionario). https://opinio.ucl.ac.uk/s?s=69395

- Los usuarios serán invitados previamente por correo electrónico o por telefóno a participar. El link será enviado por donde la persona lo indique, esto puede ser correo electrónico, mensaje de texto o Whatsapp.

- La plataforma guarda la fecha y el IP de respuesta por lo que es posible saber cuando y desde qué servidor fue contestado el cuestionario.

- En el caso de requerir asistencia telefónica, personal capacitado guiará al usuario en la completación del instrumento. En este particular caso, la llamada será grabada y registrado el consentimiento de manera oral. Esta grabación será almacenada junto a los datos personales de cada usuario.

- En el caso de requerir asistencia de interpretación, personal capacitado guiará la entrevista por videollamada. En este particular caso, la llamada será grabada y registrado el consentimiento de manera visual a través de una grabación de video con uso de lengua de señas chilena. Esta grabación será almacenada junto a los datos personales de cada usuario.

## 3. ENGLISH PROTOCOL FOR ADULTS USING CI (Short version)

PERSONAL AND AUDIOLOGICAL DATA

**1.1. Personal Data**

| Date of birth: | *(dd / mm / yyyy)* | | | FONASA | | TO |  | B |  | | C |  | D |  |  |
| --- | --- | --- | --- | --- | --- | --- | --- | --- | --- | --- | --- | --- | --- | --- | --- |
| Borough: |  | | | ISAPRE | |  | | | | | | | | | |
| Level of education: |  | Incomplete basic |  | | Primary completed | | | | | Other: | | | | | |
|  |  | Incomplete Secondary |  | | Secondary completed | | | | |  | | | | | |
|  |  | Incomplete technician |  | | Training Completed | | | | |  | | | | | |
|  |  | Incomplete university |  | | University Compleded | | | | |  |  |  |  |  |  |
|  |  |  |  | |  | | | | |  |  |  |  |  |  |

Occupation prior to hearing loss/CI:___________________________________________________________

Current occupation: __________________________________________________________________

**1.2. Family information**

| Civil Status: |  | Single | |  | Married |  | Separated | | | | |
| --- | --- | --- | --- | --- | --- | --- | --- | --- | --- | --- | --- |
|  |  | Divorced | |  | Widower |  | Partner | | | | |
| Household composition: | | |  | | | | | | | N° |  |
| Hearing Loss in the family | | | | | | | |  | Yes |  | No |
| Age-related hearing loss | | | | | | | |  | Yes |  | No |

|  |  |  |  |  |  |  |  |  |  |  |  |
| --- | --- | --- | --- | --- | --- | --- | --- | --- | --- | --- | --- |

**1.3. Audiological information**

Aetiology (e.g. to give an example) __________________________________ _____Unknown____.

Type of Hearing loss: (complete audiological diagnosis) ___________________________________.

When did your hearing loss start date: ___________________?

Was this? Sudden_____ Progressive____ Both_____.

Use of Hearing aids in the pass: Y____ N____. Where? Left_____ Right_____ Both ears____.

Current use of Hearing aids: Y____ N____. Where? Left_____ Right_____ Both ears ____.

MEDICAL AND TREATMENT HISTORY

**2.1. Medical History**

*Some doctor or health professional has told you that you have*

|  | Diabetes or blood sugar | Is it checked regularly? | |  | Yes |  | Not |
| --- | --- | --- | --- | --- | --- | --- | --- |
|  | Hypertension or high blood pressure | Is it checked regularly? | |  | Yes |  | Not |
|  | Parkinson's disease or tremors | Do you take medicine for this? | |  | Yes |  | Not |
|  | Heart problem or heart attack | Is it checked regularly? | |  | Yes |  | Not |
|  | Depression | Do you take medicine for this? | |  | Yes |  | Not |
|  | Arthritis, osteoarthritis, or joint problem | |  |  |  |  |  |
|  |  |  |  |  |  |  |  |

 Other? ___________________________________________________________________________.

Do you have any additional disability or sensorial impairment? Y___ N___ (Detail: _____________)

**2.2. Treatment information**

**Currently,** What are you receiving any treatment in relation with your CI? : Y____ N____

What type of treatment? Audiological Training____ SLT____ Psychological____ Other______

How many time per week? 1 Hr ______2 Hrs ______3 Hrs______ 4hrs or + _____

How long have you been receiving this support? 3 months_____ 6 months______ 1 year or +_____

Do you receive support from any of these professionals?

 Interpreter SLT  Audiologist  SEN Teacher  AVT Therapist  Psychologist  Other

Where? Hospital ____ Private – Paid _____Private – Free ____ Transport time from home____ min.

Frequency? ____Weekly ____Monthly_____Every 2 months_____Every 3 months or more

And at the PAST, did you receive any treatment in relation with CI or hearing aids? Y____ N____

What type of treatment? Audiological Training____ SLT____ Psychological____ Other______

How many times per week? 1 Hr ______2 Hrs ______3 Hrs______ 4hrs or + _____

How long did you receive that support? 3 months_____ 6 months______ 1 year or +_____

How long time ago? 1 year____ 3 years____ 5 years or + _____

**2.3. CI Satisfaction**

Have the results been as you expected?

**In general:** Much better___ Better ____As I thought ____No too much___Definitely Not___

**In hearing/listening**: Much better___ Better ___As I thought ___No too much___Definitely Not____

**In my occupation:** Much better___ Better ____As I thought ____No too much___Definitely Not___

Do you think that your abilities in your occupation has improved with the CI: Y____ N____?

How much the following conditions have changed after the CI?

Tinnitus: I don`t have this____. Nothing____ No too much____ Enough ____ Entirely____

Balance/Dizziness: I don`t have this____. Nothing____ No too much____ Enough ____ Entirely____

Do you think that the process offered by the hospital after your implantation was?

Very Good____ Good____ Enough_____ Poor ____ Bad

Based on your experience, how could the service provided by the hospital improve?

________________________________________________________________________________

2.4. IOI-HA PROTOCOL

2.5 Additional question

Do you think after one year of the cochlear implant, your labour status or working conditions have:

A) Diminshed

B) Mantained

C) Improved

## 4. SPANISH PROTOCOL FOR ADULTS USING CI (Extended version with IOI-HA assessment)

DATOS PERSONALES Y AUDIOLÓGICOS

**1.1. Información personal**

| Fecha de nacimiento: | *(dd / mm / aaaa)* | | | FONASA | | A |  | B |  | | C |  | D |  |  |
| --- | --- | --- | --- | --- | --- | --- | --- | --- | --- | --- | --- | --- | --- | --- | --- |
| Municipalidad : |  | | | ISAPRE | |  | | | | | | | | | |
| Nivel de educación: |  | Básico incompleto |  | | Básica completa | | | | | Otra: | | | | | |
|  |  | E. Media incompleta |  | | E.Media completa | | | | |  | | | | | |
|  |  | Técnico incompleto |  | | Tecnico/Superior completo | | | | |  | | | | | |
|  |  | Universidad incompleta |  | | Universidad completa | | | | |  | | | | | |
|  |  |  |  |  |  |  |  |  |  |  |  |  |  |  |  |

Ocupación antes de la pérdida auditiva / CI: _____________________________________________

Ocupación actual: __________________________________________________________________

**1.2. Información familiar**

| Estado civil: |  | Soltero/a | |  | Casado/a |  | Separado | | | | |
| --- | --- | --- | --- | --- | --- | --- | --- | --- | --- | --- | --- |
|  |  | Divorciado/a | |  | Viudo/a |  | Conviviente | | | | |
| Composición del hogar (Quienes): | | |  | | | | | | | N ° |  |
| Pérdida auditiva en la familia | | | | | | | |  | si |  | No |
| Pérdida auditiva relacionada con la edad | | | | | | | |  | si |  | No |
|  |  |  |  |  |  |  |  |  |  |  |  |

|  |  |  |  |  |  |  |  |  |  |  |  |
| --- | --- | --- | --- | --- | --- | --- | --- | --- | --- | --- | --- |

**1.3. Información audiológica**

Causa de la Hipoacusia (Ej; hereditaria, por edad, etc) _______________________Desconocido____.

Tipo de hipoacusia: (diagnóstico audiológico si lo conoce) __________________________________.

¿Cuándo comenzó su pérdida auditiva (año/mes): ___________________?

¿Su inicio fue? Súbito_____ Progresivo____ Ambos_____.

¿Usaba de audífonos antes del IC?:

Si____ N____. ¿Dónde? Izquierda___ Derecha___ Ambos oídos___.

¿Usa audífonos actualmente?:

S____ N____. ¿Dónde? Izquierda_____ Derecha_____ Ambos oídos ____.

HISTORIAL MÉDICO Y DE TRATAMIENTOS

**2.1. Historial médico**

*Algún médico o profesional de la salud le ha dicho que tiene*

|  | Diabetes o azúcar en sangre | ¿Se controla con regularidad? | |  | si |  | No |
| --- | --- | --- | --- | --- | --- | --- | --- |
|  | Hipertensión o presión arterial alta | ¿Se controla con regularidad? | |  | si |  | No |
|  | Enfermedad de Parkinson o temblores | ¿Toma medicamentos para esto? | |  | si |  | No |
|  | Problema cardíaco o ataque cardíaco previo | ¿Se controla con regularidad? | |  | si |  | No |
|  | Depresión | ¿Toma medicamentos para esto? | |  | si |  | No |
|  | Artritis, osteoartritis o problema articular | |  |  |  |  |  |
|  |  |  |  |  |  |  |  |

¿Otro? ___________________________________________________________________________.

¿Tiene alguna discapacidad adicional o deficiencia sensorial? S___ N___ (Detalle: _____________)

**2.2. Información de tratamiento**

**Actualmente,** ¿Está recibiendo tratamiento/terapia en relación con su IC? : S____ N____

¿Qué tipo de tratamiento?

Entrenamiento auditivo____ Fonoaudiología____ Psicológia____ Otro______

¿Cuantas veces por semana? 1 hora ______2 horas ______3 horas______ 4 horas o + _____

¿Cuánto tiempo llevas recibiendo este apoyo? 3 meses_____ 6 meses______ 1 año o + _____

¿Recibe apoyo de alguno de estos profesionales?

  ÿ Intérprete ÿ Fononoaudiólogoÿ Audiólogo ÿ Docente ÿ  Psicólogo ÿ Otro

¿Dónde?

Hospital ____ Privado - Pagado _____ Privado - Gratis ____ Tiempo de transporte desde casa____ min.

¿Frecuencia? ____ Semanalmente ____ Mensualmente_____ Cada 2 meses_____ Cada 3 meses o más

**Y en el PASADO,** ¿recibió algún tratamiento en relación al Implante Coclear o audífonos? S____ N____

¿Qué tipo de tratamiento? Fonoaudiología/Audiología____ Psicología____ Otro______

Cuantas veces por semana 1 hora ______2 horas ______3 horas______ 4 horas o + _____

¿Cuánto tiempo recibió ese apoyo? 3 meses_____ 6 meses______ 1 año o + _____

¿Hace cuanto tiempo? 1 año____ 3 años____ 5 años o + _____

**2.3. Satisfacción de CI**

¿Los resultados han sido los esperados?

**En general:**

Mucho mejores___ Mejores____ Como pensaba ____ No tanto ___ Definitivamente no___

**Al oír / escuchar** :

Mucho mejores___ Mejores___ Como pensaba ___ No tanto___ Definitivamente no____

**En mi ocupación:**

Mucho mejores___ Mejores ____ Como pensaba ____ No tanto___ Definitivamente no___

¿Cree que sus habilidades en su ocupación han mejorado con el IC: Y____ N____?

¿Cuánto han cambiado las siguientes condiciones después de la IC?

Tinnitus (pitidos en los oídos):

No tengo tinnitus____ Nada____ No demasiado____ Suficiente ____ Bastante____Totalmente____

Equilibrio / mareos:

No tengo mareos____ Nada____ No demasiado____ Suficiente ____ Bastante____Totalmente____

¿Crees que el proceso que te ofreció el hospital tras tu implantación fue?

Muy bueno____ Bueno____ Suficiente_____ Malo ____ Muy Malo

Según su experiencia, ¿cómo podría mejorar el servicio que brinda el hospital?

__________________________________________________________________________________

1. Piense cuanto ha utilizado usted su implante coclear en las últimas dos semanas. En un día común ¿Cuantas horas ha usado el implante coclear?

- Ninguna
- Menos de una hora al día
- De 1 a 4 horas al día
- De 4 a 8 horas al día
- Más de 8 horas al día

2. Piense en una situación donde usted realmente hubiera querido escuchar mejor antes de obetener su implante coclear actual. En las últimas semanas ¿cuanto le ha ayudado el implante coclear en esa situación?

- No ayudó
- Ayudó poco
- Ayudó moredamente
- Ayudó bastante
- Ayudó mucho

3. Piense una vez más en la situación en la cual usted realmente desea escuchar mejor. Cuando utiliza el implante actual ¿Cuanta dificultad tiene todavía en esa situación?

- Mucha dificultad
- Bastante dificultad
- Moderada dificultad
- Poca dificultad
- Ninguna dificultad

4. Considerando todo. ¿Piensa usted que su implante actual vale la pena?

- No vale la pena
- Vale poco la pena
- Vale la pena moderadamente
- Vale la pena bastante
- Vale la pena mucho

5. En las últimas dos semanas, con el implante que utiliza actualmente. ¿Cuanto ha afectado su rutina diaria su dficultad auditiva?

- Afectado mucho
- Afectado bastante
- Afectado moderadamente
- Afectado poco
- Afectado nada

6. En las dos semanas pasadas, con su implante ¿Cuánto piensa usted que su dificultad auditiva ha molestado a otras personas?

- Molestado mucho
- Molestado bastante
- Molestado moderadamente
- Molestado poco
- Molestado nada

7. Considerando todo ¿Cuánto ha cambiado su manera de disfrutar la vida con el implante que utiliza actualmente?

- Empeoró
- Sin cambio
- Mejoró poco
- Mejoró bastante
- Mejoró mucho

8. Usted cree que luego de un año de uso del implante coclear sus condiciones laborales o de trabajo han:

A) Disminuido

B) Sido iguales

C) Mejorado

**S2 Appendix – Consort diagram**


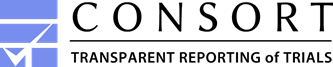


**Enrolment**

**Analysis**

Analysed **(n= 79 )**

• Excluded from analysis (n= 0 )

Excluded **(n= 44 )**

- Not meeting inclusion criteria (n= 3)
- Declined to participate (n= 0)
- Other reasons (n=41 )

Assessed for eligibility **(n=123)**
